# Association Between Pandemic-Related Maternal Perinatal Anxiety and Early Postpartum Breastfeeding Success

**DOI:** 10.7759/cureus.57590

**Published:** 2024-04-04

**Authors:** Neslihan Bezirganoğlu Altuntaş, Sema Baki Yıldırım, Huri Güvey, Yesim Bayoglu Tekin

**Affiliations:** 1 Obstetrics and Gynecology, Trabzon Kanuni Training and Research Hospital, Trabzon, TUR; 2 Obstetrics and Gynecology, Giresun University, Giresun, TUR; 3 Obstetrics and Gynecology, Kütahya Parkhayat Hospital, Kütahya, TUR

**Keywords:** stai score, cas score, latch score, breastfeeding, covid-19 pandemic

## Abstract

Background and objectives: Prior studies have shown conflicting results on the impact of maternal anxiety on breastfeeding initiation and success. Furthermore, a substantial increase in maternal anxiety levels was shown in response to the COVID-19 pandemic. In this study, we aimed to investigate the relationship between maternal perinatal anxiety induced by the COVID-19 pandemic and early breastfeeding outcomes.

Materials and methods: This cross-sectional study was conducted in two regional maternity hospitals, involving 220 first-time pregnant patients with a gestational age of ≥37 weeks. All patients had no current diagnosis of COVID-19 and no cases of COVID-19 in their close environment at the time of admission. At 24-48 hours postpartum or at the time of discharge, three following scoring systems were employed: the Coronavirus Anxiety Scale (CAS), the State-Trait Anxiety Inventory (STAI), and the LATCH (short for latch, audible swallowing, type of nipple, comfort, and hold) score. A LATCH score of ≥8 was chosen as the cutoff point for defining successful breastfeeding performance. Spearman's rank correlation was used to evaluate relationships between the CAS, STAI scores, maternal and infant factors, and LATCH scores.

Results: There were no differences in baseline characteristics between groups categorized as successful and unsuccessful in breastfeeding initiation. The mean total STAI score was 86.3±13.2, the CAS score was 1.07±1.91, and the LATCH score was 8.42±1.7. Although there was an increase in State-Trait Anxiety Inventory-State Anxiety (STAI-S) scores compared to State-Trait Anxiety Inventory-Trait Anxiety (STAI-T) scores, and the STAI-S score and CAS score were higher in the unsuccessful group, these differences did not reach statistical significance (p = 0.22 and 0.16, respectively). When we evaluated the correlation of the LATCH score with STAI total, STAI-S and STAI-T scores, CAS score, and maternal and infant factors, only the type of delivery showed a significant correlation with the LATCH score (p = 0.008).

Conclusions: Early postpartum breastfeeding efficiency, as measured by the LATCH score, was only correlated with the type of delivery. No significant correlation was found between pandemic-related maternal perinatal anxiety and early postpartum breastfeeding success.

## Introduction

The emergence of COVID-19 in Wuhan, China, transformed into a global public health crisis, significantly impacting vulnerable populations, including pregnant and breastfeeding women [1]. The peripartum period is characterized by substantial hormonal fluctuations, making women more susceptible to mental health challenges. Previous studies have shown that peripartum women showed a higher susceptibility to acquiring psychiatric disorders, including depression and anxiety, during pregnancy, with the prevalence of anxiety disorders in this period reaching up to 25% in developing countries [2-4]. Consequently, the COVID-19 pandemic, in addition to its physical effects, has presented a psychological strain to pregnant and postpartum women, as evidenced by studies showing a substantial increase in maternal anxiety levels in response to the pandemic [5].

One of the main concerns that emerged among postpartum women during the COVID-19 pandemic included the potential risk of vertical transmission of SARS-CoV-2 through breastfeeding [6]. Initial investigations identified the presence of SARS-CoV-2 antigens in human breast milk; however, a consistent consensus was absent, as certain subsequent studies failed to replicate these observations. [7-9]. The anxiety amplified by the COVID-19 pandemic itself, coupled with the ongoing debate over the safety of breast milk, may further influence the breastfeeding performance of postpartum women during this critical period. 

The early postpartum period is crucial for assessing breastfeeding performance and identifying any potential issues. To facilitate this evaluation, several scoring systems have been developed [10]. One such widely utilized scoring system is the Latch Breastfeeding Assessment Tool (LBAT), originally introduced by Jensen et al. in 1994. The LBAT has proven to be reliable and efficient, serving as a predictor for exclusive breastfeeding and long-term success [11].

There is a scarcity of existing literature that investigates the influence of pandemic-related anxiety on lactation performance [12]. Therefore, our study's primary objective was to evaluate the relationship between COVID-19 pandemic-induced maternal perinatal anxiety and early breastfeeding success using the LATCH (short for latch, audible swallowing, type of nipple, comfort, and hold) score. 

## Materials and methods

This cross-sectional study was conducted between August 2020 and June 2021 at two maternity hospitals, namely, Kanuni Education and Research Hospital, Trabzon, Türkiye, and Giresun University Maternity and Children’s Hospital, Giresun, Türkiye. All pregnant women who were admitted to the antenatal outpatient clinic of these two hospitals and subsequently hospitalized for delivery were offered to participate in the survey if they met the following criteria: gestational age ≥37 weeks at the time of admission, first-time pregnancy, expected fetal weight >2,500 g, absence of additional comorbidity, maternal age between 18 and 40 years old, no current diagnosis of COVID-19, and no cases of COVID-19 from their close environment at the time of admission.

Pregnant women who did not meet these criteria were excluded from the study. Two hundred twenty patients who met the inclusion criteria and consented to participate were enrolled in the study. All participants signed a written informed consent form at enrollment. The study was conducted in accordance with the Declaration of Helsinki and approved by the ethics committee of Kanuni Education and Research Hospital, Trabzon, Türkiye (2020/58).

All participants underwent a comprehensive physical examination at the time of admission, and an ultrasound assessment was performed. We closely monitored and followed the participants from the time of enrollment until delivery. Sociodemographic data, including maternal age, gestational age, mode of conception, type of delivery, birth weight of the infant, and the level of maternal education, were recorded. At 24-48 hours postpartum or at the time of discharge, the following three scoring systems were employed: the Coronavirus Anxiety Scale (CAS), the State-Trait Anxiety Inventory (STAI), and the LBAT.

Measures

Coronavirus Anxiety Scale (CAS)

The CAS scoring system was developed by Lee in 2020 to identify cases of dysfunctional anxiety related to COVID-19 [13]. It consists of five Likert-type questions. The scoring system for the CAS is as follows: “0”: not at all; “1”: rare, less than a day or two; “2: several days; “3”: more than seven days; “4”: nearly every day over the last two weeks. When the total score is equal to or higher than nine, it indicates a likelihood of dysfunctional anxiety. The Cronbach’s alpha coefficient of the scale was found to be 0.918 in Lee’s study and 0.778 in the present study.

State-Trait Anxiety Inventory (STAI)

The STAI is a self-rating scale developed by Speilberger et al. in 1979 [14]. It includes 40 Likert-type questions, which were scored on a scale ranging from one to four. Higher scores on the STAI indicate a higher level of anxiety, with scores above 40 suggesting the presence of anxiety. The scale consists of two distinct subscales: State-Trait Anxiety Inventory-Trait Anxiety (STAI-T) evaluates a person's general propensity to experience anxiety across time and circumstances, while State-Trait Anxiety Inventory-State Anxiety (STAI-S) measures their anxiety in the present situation. The reliability and validity of STAI in Turkish were provided by Öner [15]. The Cronbach’s alpha coefficient of the scale was calculated to be 0.848 in the present study.

Latch Breastfeeding Assessment Tool

The LBAT describes the establishment and development of the act of breastfeeding and helps to indicate breastfeeding problems and areas where the mother needs support. It is based on five different categories, including the infant's attachment to the breast, the number of swallows, the nipple type, maternal comfort, and the amount of assistance that the mother requires to hold her baby to her breast (Table 1) [11]. Each item was scored between 0 and two. The measurement tool does not have a predetermined cut-off point; instead, it correlates higher LATCH scores with higher success in breastfeeding. However, several studies have shown the LATCH score cutoff of ≥8 at 48 hours and/or discharge had the highest sensitivity and specificity in predicting exclusive breastfeeding [16,17]. In the present study, a LATCH score of ≥8 was chosen as the cutoff point for defining successful breastfeeding performance.

**Table 1:** The LATCH score LATCH: latch, audible swallowing, type of nipple, comfort, and hold [11]

		0	1	2
L	Latch	Too sleepy or reluctant	Repeated attempts for sustained latch or suck, hold the nipple in the mouth, and stimulate to suck	Grasps breast, tongue down, lips flanged, and rhythmic sucking
A	Audible swallowing	None	A few with stimulation	Spontaneous and intermittent <24 hours old; Spontaneous and frequent >24 hours old
T	Type of nipple	Inverted	Flat	Everted (after stimulation)
C	Comfort (breast/nipple)	Engorged, cracked, bleeding, large blisters or bruises, and severe discomfort	Filling reddened/small blisters or bruises, mild/moderate discomfort	Soft non-tender
H	Hold (positioning)	Fully assisted	Minimal assistance (i.e. elevate the head of the bed or place pillows for support); teach one side and the mother does the other; the staff holds and then the mother takes over.	No assistance from the staff; the mother is able to position/hold the infant

Statistical analysis

All analyses were performed on IBM SPSS Statistics for Windows, version 23 (IBM Corp., Armonk, NY). Categorical variables were described as percentages, and for continuous variables, mean and standard deviation (SD) were given as descriptive variables. Abnormally distributed data were evaluated with the Kolmogorov-Smirnov test. Univariate analysis was performed for the demographic characteristics and anxiety scores of the patients to predict breastfeeding initiation success by using the Mann-Whitney U-test and chi-square test as appropriate for individual variables. The LATCH score of ≥8 was chosen as the cutoff point for defining successful breastfeeding performance. Pearson's or Spearman's rank correlation coefficients were calculated to evaluate relationships between the CAS, STAI scores, maternal and infant factors, and LATCH scores, depending on the normality of the distribution. A p-value of <0.05 was considered statistically significant. The sample size was based on effect size = 0.2, 0.05 of alpha (α) error, and 0.80 of power. The calculated sample size was 158.

## Results

Among the 278 women recruited for the study, 45 couldn’t complete the questionnaire, and 13 refused to participate. Consequently, we reviewed a total of 220 pregnant women who met the inclusion criteria. The mean age of participants was 26.08 ±4.01 years, and the gestational age was 39.2 ±1.09 weeks. While 129 pregnant women gave vaginal birth, 41.4% of participants underwent a cesarean section. There were no differences in the baseline characteristics between the groups categorized as successful and unsuccessful in breastfeeding initiation, as determined by the LATCH score (Table 2).

**Table 2:** Characteristics of the patients (mothers and infants) Data are given as mean ± standard deviation for continuous variables and as frequency (percentage) for categorical variables. ^a^ p<0.05 values were considered as significant LATCH: latch, audible swallowing, type of nipple, comfort, and hold

		Total cohort (n=220)	LATCH score <8 (n=49)	LATCH score ≥8 (n=171)	p-value^a^
Maternal age (years) (SD)		26.08 ±4.01	25.75±4.11	26.18 ± 3.99	0.57
Gestational age, (weeks) (SD)		39.2±1.09	39.2±1.46	39.3±1.38	0.58
Infant weight (grams) (SD)		3361±435	3323± 497	3373±417	0.54
Mode of conception, n (%)	Spontaneous	202 (91.8)	44 (89.7)	158 (92.3)	0.84
	Assisted reproductive technique	18 (8.2)	5 (10.3)	13 (7.7)	
Type of birth, n (%)	Vaginal birth	129 (58.6)	27 (55.1)	102 (59.6)	0.85
	Cesarean section	91 (41.4)	22 (44.9)	69 (40.4)	
Education status, n (%)	Illiterate	1 (0.5)	1 (2)	0 (0)	0.24
	Secondary school	39 (17.7)	7 (14.4)	32 (18.5)	
	High school	81 (36.8)	20 (41.8)	61 (35.5)	
	University	99 (45)	20 (41.8)	78 (46)	
Marital status, n (%)	Married	216 (98.2)	48 (98)	168 (98.2)	0.62
	Single	4 (1.8)	1 (2)	3 (1.8)	
Perceived income level, n (%)	Poor	18 (8.2)	3 (6.1)	15 (8.8)	0.78
	Moderate	188 (85.5)	43 (87.8)	145 (84.8)	
	Good	14 (6.3)	53 (6.1)	11 (6.4)	

The mean total STAI score was 86.3±13.2, the CAS score was 1.07±1.91, and the LATCH score was 8.42±1.7. One hundred fifty-one patients (68.8%) had STAI-S score equal to or higher than 40. Although there was an increase in STAI-S scores compared to STAI-T scores and the STAI-S score and CAS score were higher in the unsuccessful group, the difference was not statistically significant (p = 0.22 and 0.16, respectively) (Table 3).

**Table 3:** Comparison of CAS and STAI scores based on breastfeeding success STAI: State-Trait Anxiety Inventory, CAS: Coronavirus Anxiety Scale; LATCH: latch, audible swallowing, type of nipple, comfort, and hold Data are given as mean ± standard deviation  for continuous variables ^a^ p<0.05 values were considered as significant

		Total cohort (n=220)	LATCH score <8 (n=49)	LATCH score ≥8 ( n=171)	p-value^a^
STAI score	State score	44.68±9.37	46.53±10.87	44.38±8.74	0.22
	Trait score	41.7±6.6	41.67±7.50	41.82±6.47	0.90
	Total score	86.3±13.2	88.19±14.38	86.07±12.91	0.39
CAS score		1.07±1.91	1.47±2.71	0.96±1.65	0.16

No patient had a ≥9 CAS score. When we evaluated the correlation of the LATCH score with STAI total, STAI-S and STAI-T scores, CAS score, and maternal and infant factors, only the type of delivery had a significant correlation with the LATCH score (p = 0.008). Vaginal birth was positively associated with successful early postpartum breastfeeding performance; however, the correlation coefficient was weak (r = 0.209) (Table 4).

**Table 4:** Correlation of breastfeeding success with STAI score, CAS score, and maternal and infant factors STAI: State-Trait Anxiety Inventory, CAS: Coronavirus Anxiety Scale; LATCH: latch, audible swallowing, type of nipple, comfort, and hold r: Correlation coefficient; Spearman's correlation tests were applied a p<0.05 values were considered as significant

		LATCH score	
		r	p-value^a^
Maternal age		0.08	0.92
Education status		0.03	0.70
Type of birth		0.209	0.008
Infant weight		0.08	0.31
STAI score	State score	0.086	0.73
	Trait score	0.016	0.74
	Total score	0.03	0.96
CAS score		0.096	0.23

## Discussion

In this study, we evaluated the anxiety levels of pregnant women during the COVID-19 pandemic and investigated the potential impact of elevated maternal perinatal anxiety and depression symptoms on early postpartum breastfeeding success. Based on our results, over half of the participants had a STAI-S score of 40 or higher during the COVID-19 pandemic. However, none of the participants had a CAS score higher than nine. Furthermore, early postpartum breastfeeding efficiency, as measured by the LATCH score, was only correlated with the type of delivery. No significant correlation was found with the STAI-S and STAI-T scores, the CAS score, or other maternal and infant factors.

During the ongoing COVID-19 pandemic, previous studies have consistently reported an increase in anxiety symptoms among pregnant women [18,19]. Lebel et al. observed a significant rise in anxiety symptoms compared to pre-pandemic pregnancy cohorts, with 43.6% of participants experiencing moderately elevated anxiety symptoms and 10.3% reporting severely elevated anxiety symptoms [18]. In line with those findings, another study showed higher STAI-S scores in pregnant women admitted for delivery during the pandemic prior to the outbreak [19]. However, in that study, STAI-T scores did not demonstrate statistical significance. The authors concluded that the change in STAI-S scores among pregnant women was due to their fear of COVID-19. Both Hoşoglu et al. and Mappa et al. found an increase in STAI-T and STAI-S scores of pregnant women during the COVID-19 pandemic and a positive linear correlation between these scores [20,21]. In our study, 68.8% of the patients had elevated STAI-S scores, and the mean STAI-S scores were higher than the STAI-T scores. Although we believe fear of COVID-19 significantly contributed to these outcomes, none of the patients had dysfunctional anxiety solely attributed to the COVID-19 pandemic based on CAS scores in our cohort.

It is expected that women experiencing postpartum anxiety and/or depression are less likely to achieve exclusive breastfeeding success and may be more prone to prematurely discontinuing breastfeeding. However, recent studies don’t support this negative association [22]. Furthermore, a systematic review revealed no association between prenatal anxiety and exclusive breastfeeding or breastfeeding initiation [23]. Consistent with these recent findings, the results of our study revealed that the CAS score and the STAI-S score of the participants had no impact on early breastfeeding outcomes. Nevertheless, few studies in the literature yielded different results. Despite the absence of high CAS scores in their study similar to ours, Ataman Bor et al. observed a negative effect on breastfeeding related to CAS scores [24]. In addition, a web-based survey involving 849 pregnant women also found that mothers' anxiety and confusion related to COVID-19 had a negative impact on breastfeeding self-efficacy [25]. The discrepancy between the outcomes of our study and other studies could be attributed to variations in the characteristics of the study populations and breastfeeding evaluation. Our study intentionally excluded participants with COVID-19 and focused solely on first-time mothers without prior COVID-19 history within the initial 48 hours of the postpartum period. Moreover, we determined the LATCH score through direct observation by a healthcare provider, in contrast to the breastfeeding self-efficacy scale.

Cesarean deliveries have been linked to unsuccessful initiation of breastfeeding and decreased milk production, potentially due to decreased oxytocin secretion or maternal stress [26]. Moreover, vaginal delivery is consistently associated with increased breastfeeding rates and success [27]. Our results also showed a correlation between LATCH score and type of delivery, indicating that the vaginal delivery group had better LATCH scores. Previous studies have reported the positive impact of cognitive-behavioral counseling and practical support for breastfeeding methods by trained healthcare providers in the first days of life on breastfeeding success [28]. Since no participants received professional counseling on stress management during the perinatal period in this study, the effectiveness of counseling is beyond the scope of this study. Our study has other limitations. First, we evaluated the rise of anxiety related to the COVID-19 pandemic but did not further detail additional factors contributing to the rise of perinatal anxiety. Second, we investigated lactation performance only in the first 24-48 hours of the postpartum period, so we could not determine how these symptoms and scores change in the subsequent weeks or months of the postpartum period.

## Conclusions

To our knowledge, this study represents one of the first studies in the literature evaluating the effect of COVID-19-related anxiety on the early postpartum LATCH score. Contrary to our initial hypothesis, the LATCH score of participants, which measures the success of breastfeeding initiation, showed no correlation with the STAI score, CAS score, or maternal and infant factors. These results require future studies with larger samples of patients for a comprehensive evaluation of maternal anxiety on breastfeeding success.
